# Superior Temperature-Dependent Mechanical Properties and Deformation Behavior of Equiatomic CoCrFeMnNi High-Entropy Alloy Additively Manufactured by Selective Laser Melting

**DOI:** 10.1038/s41598-020-65073-2

**Published:** 2020-05-15

**Authors:** Young-Kyun Kim, Sangsun Yang, Kee-Ahn Lee

**Affiliations:** 1 Department of Materials Science and Engineering, Inha University, Incheon, 22212 Republic of Korea; 2 Korea Institute of Materials Science (KIMS), Changwon, 51508 Republic of Korea

**Keywords:** Engineering, Materials science

## Abstract

The microstructure, temperature-dependent mechanical properties and deformation behaviors of equiatomic CoCrFeMnNi high-entropy alloy (HEA) additively manufactured by selective laser melting (SLM) were investigated. SLM-built HEA had a face-centered cubic (FCC) single-phase random solid solution. In addition, SLM-built HEA was composed of epitaxial growth grains, dislocation network and nano-sized oxides. Room- and high-temperature compression tests confirmed that SLM-built HEA has outstanding mechanical properties in all temperature ranges compared to equiatomic CoCrFeMnNi HEAs reported up to the present. The excellent mechanical properties of SLM-built HEA were achieved with fine grains, high dislocation density and fine precipitates at low temperatures (25 °C to 600 °C), and by high dislocation density and fine precipitates at high temperatures (700 °C or higher). On the other hand, the deformation microstructure showed that slip and deformation twins are the main deformation mechanisms from 25 °C to 600 °C, and slip and partial recrystallization are the main deformation mechanisms above 700 °C. Based on the above findings, this study also discusses correlations among the microstructure, superior mechanical properties and deformation mechanisms of SLM-built equiatomic CoCrFeMnNi HEA.

## Introduction

Unlike other conventional alloys based on a “principal element,” high-entropy alloy (HEA) is a unique material that maintains a random solid solution structure without forming intermetallic compounds in operating temperatures due to its high configurational entropy^1–3^. It also has outstanding physical and chemical properties, which makes it a potential replacement for traditional metallic materials, and it has recently been referred to as a multi-principal element alloy (MPEA) or compositionally concentrated alloy (CCA)^4–9^.

As attention in HEAs increases, many studies have been conducted to explore its mechanical, physical and chemical properties^10–15^. In particular, equiatomic CoCrFeMnNi HEA, which was the first developed HEA, is the most studied because of its excellent combination of tensile strength and elongation along with excellent fracture toughness and corrosion resistance^6,16–21^. However, despite the advantages of equiatomic CoCrFeMnNi HEA, many issues arise owing to its low yield strength and costly and complex thermomechanical processing (i.e., multiple casting → hot rolling → homogenization → cold rolling → annealing → cutting, etc.). Therefore, there are studies that have suggested the application of oxide dispersion strengthening or manufacturing of non-equiatomic HEA^22,23^, and there have been recent attempts to improve the properties of HEA by adding interstitial atoms^24^. However, the alloys in these studies also underwent various complex thermomechanical processing routes, so excessive material and time would be consumed to manufacture an actual product. As a result of this, new methods of manufacturing HEA are being suggested, and among them, the additive manufacturing (AM) technique, which is capable of manufacturing near net shape products, is being considered as an alternative^25,26^.

AM is an emerging new technology by which components are manufactured in a layer-by-layer manner based on a computer-aided design (CAD) model to create a complex three-dimensional shape^27,28^. Unlike conventional manufacturing processes (casting, machining, etc.), which have shape limitations, AM has almost no geometric constraints, which allows the manufacturing of a variety of complex shapes^29,30^. Because of this characteristic, it is possible to manufacture components that were difficult to manufacture before, as well as high-performance parts for use in aerospace, defense, automotive and biomedical fields. Since the selective laser melting (SLM) process offers a fast cooling rate among the metal AM processes, it is possible to improve the mechanical properties along with achieving an excellent dimensional accuracy^31,32^. Due to these advantages, SLM is one of the most studied AM techniques at present, and SLM is expected to be the most suitable method for manufacturing HEA.

In our previous study^33^, we confirmed that SLM-built equiatomic CoCrFeMnNi HEA has outstanding room temperature mechanical properties. Furthermore, equiatomic CoCrFeMnNi HEA is known to have high phase stability, which will allow it to be used in high temperatures^34^. However, despite the possibility of its use as a structural material in high temperature or extreme environments, studies conducted on AM-processed equiatomic CoCrFeMnNi HEA have been limited to fundamental level investigations such as manufacturing, microstructural analysis and room temperature tensile properties^35–40^. Furthermore, there have been no studies that investigated the influence of the unique microstructure of SLM-built HEA on high-temperature mechanical properties and deformation mechanism.

In this study, equiatomic CoCrFeMnNi HEA, which has outstanding phase stability, was manufactured using SLM, and its temperature-dependent mechanical properties were investigated. Also, this study attempted to identify the room- and high-temperature deformation mechanisms of SLM-built equiatomic CoCrFeMnNi HEA in relation to its unique microstructure.

## Results

### Microstructure of selective laser-melted equiatomic CoCrFeMnNi high-entropy alloy

**Figure 1:**
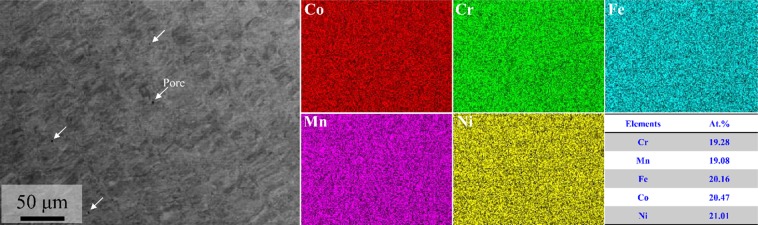
Raw equiatomic CoCrFeMnNi HEA pre-alloyed powders: (**a**) SEM morphology, (**b**) SEM-EDS mapping results, and (**c**) particle size distributions.

Figure 1 shows an SEM-BSE image and EDS mapping results of as-built equiatomic CoCrFeMnNi HEA. The BSE image revealed some micron-scale gas pores, but coarse defects or cracks were not observed in the as-built HEA (Porosity: 0.13%). The density of the present alloy was measured as 7.96 g/cm^3^ which was similar to that of the theoretical density of equiatomic CoCrFeMnNi HEA. In addition, Co, Cr, Fe, Mn, and Ni were all around 20 at. % in EDS mapping analysis, and all elements were evenly distributed. Li *et al*.^38^ found that Mn undergoes segregation when equiatomic CoCrFeMnNi HEA is manufactured using SLM. According to Wang *et al*.^41^, when equiatomic CoCrFeMnNi HEA was manufactured with pre-alloyed powder using electron beam melting (EBM), it was confirmed that Fe, Cr and Co elements segregate into the dendrite, whereas, Mn and Ni segregate into the interdendrite region. However, when the optimal process condition using SLM was deduced, no dendritic microstructure was formed, and it was possible to manufacture a random solid solution HEA with compositional homogeneity.

**Figure 2:**
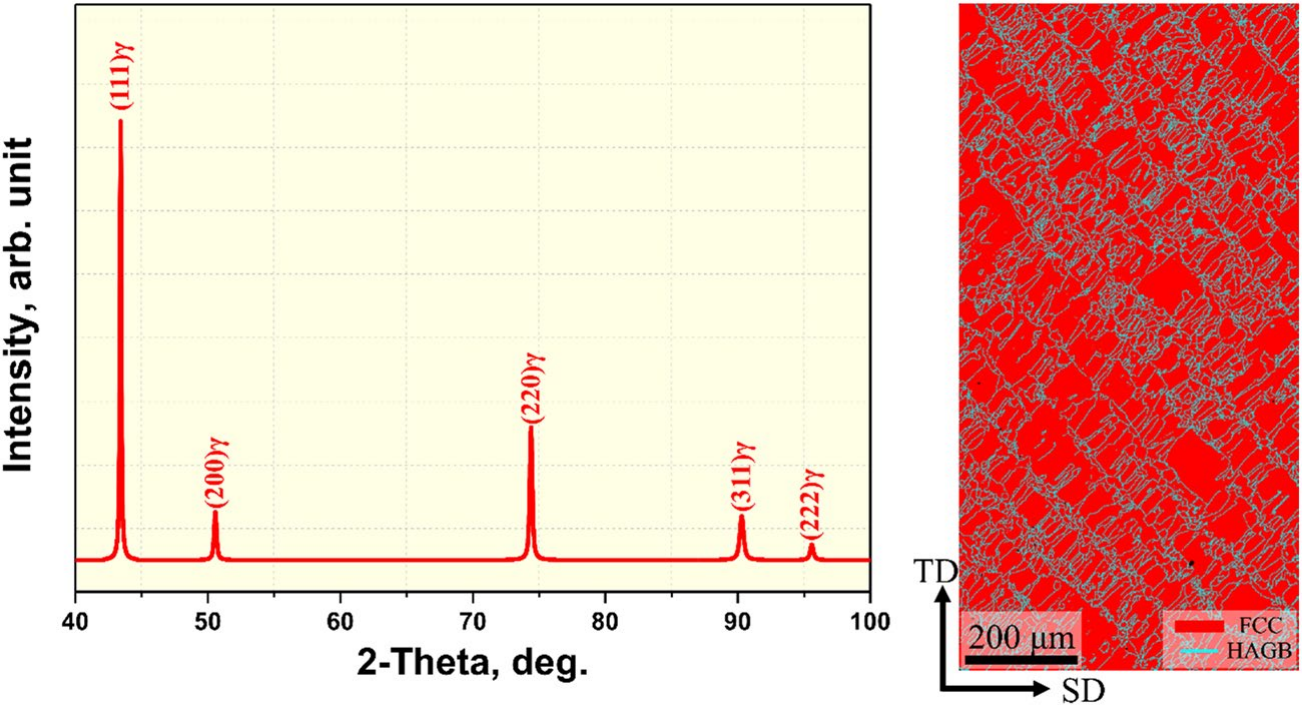
Elemental distribution analysis results of as-built equiatomic CoCrFeMnNi high-entropy alloy using BSE-EDS mapping.

Figure 2 shows the X-ray diffraction spectra (a) and electron backscatter diffraction (EBSD) phase map (b) of as-built equiatomic CoCrFeMnNi HEA. In the XRD analysis, only FCC single phase was found, and in the EBSD phase map, FCC single phase was also detected. From these findings it was possible to confirm that no impurities or inclusions were formed during the SLM process. Also, average dislocation density was calculated from the XRD pattern using convolutional multiple whole profile (CMWP) analysis. The SLM-built HEA has a higher dislocation density of 1.88 × 10^14^ m^−2^ as compared to the homogenized HEA^33^. This is a common tendency found in AM-processed materials, and it is known to be caused by thermal contraction strain that occurs during rapid solidification and cooling^42^. Linear thermal strain ($\triangle {\rm{\varepsilon }}$) during cooling is approximately 0–2%, and the thermal contraction stress occurring at this stage can form a large number of dislocations.

**Figure 3:**
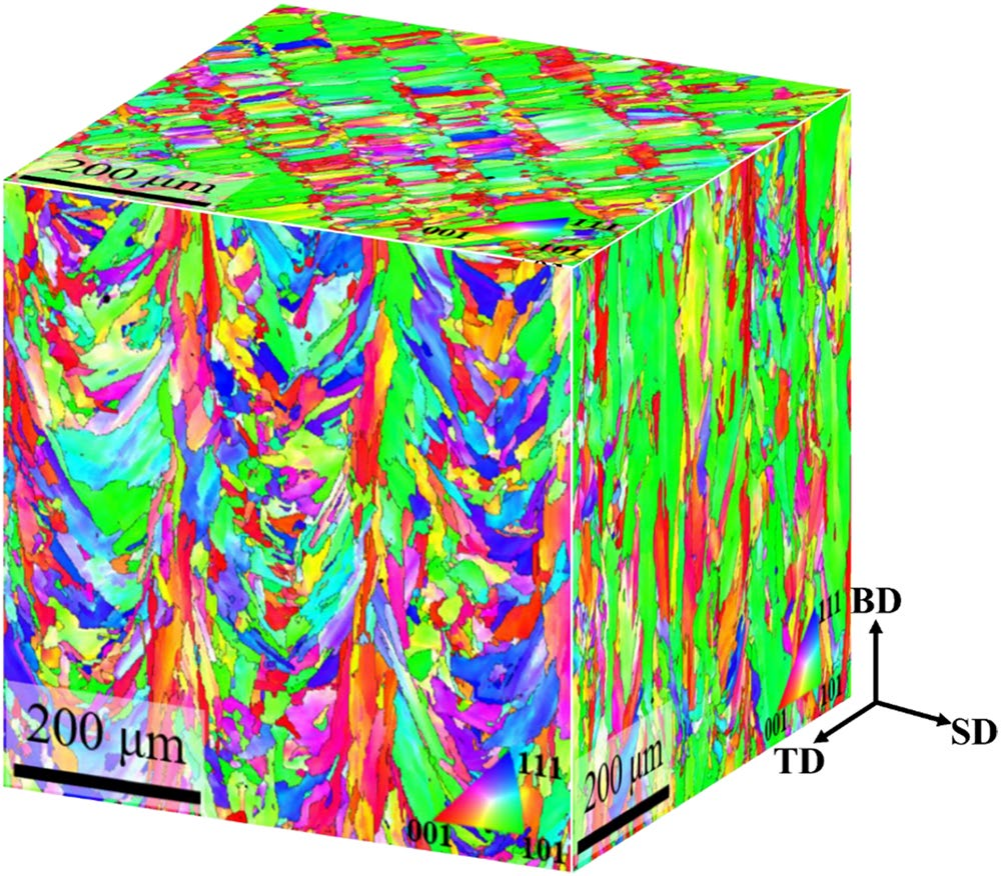
(**a**) X-ray diffraction patterns and (**b**) EBSD phase map of as-built sample.

Figure 3 shows three-dimensional EBSD inverse pole figure (IPF) maps of as-built HEA. In the EBSD IPF maps, epitaxial growth grains were observed in the planes perpendicular to the transverse direction and scanning direction (hereinafter referred to as TD plane and SD plane) to the building direction (BD). On the other hand, ~80 μm traces were found in the case of the BD plane, and as it was a similar size as the hatch space, it was possible to identify it as the laser track boundary. In other words, completely different grain structures were formed according to the direction. The reason for this phenomenon is that the grains grow in the direction of heat release during AM, and thus it is considered that epitaxial growth appears as heat is released to BD in the TD and SD planes. The average grain size of the BD, TD and SD planes measured 5.98, 12.93 and 15.66 μm, respectively.

Meanwhile, AM materials are known to develop a strong <001> texture along the BD, and this is mainly due to <001> having a tendency to develop in the direction of thermal dissipation in FCC structures^43^. However, as shown in Fig. 3, the present alloy developed strong <001> texture as well as <101> and <111> textures. After conducting pole figure analysis of BD, the maximum intensity of multiples of uniform density (MUD) measured 10.12, 4.52 and 6.70, respectively, in <001 > , <101> and <111 > (Fig. S1). In other words, in addition to <001> texture, <101> and <111> textures were developed. In relation to this, many recent studies reported the formation of <101> texture rather than <001> texture along the BD^44–47^. Wang *et al*.^47^. confirmed that as strut thickness decreased, <101> texture transformed into <001> texture in 316 L, a typical FCC alloy, manufactured with PBF. The reason for this is that the path that dissipates heat (metal powder) becomes narrower as the strut thickness decreases, which in turn results in the development of strong <001> texture. Sun *et al*.^48^ analyzed texture evolution by changing scanning strategies with SLM, and as a result, reported that strong <101> texture and weak <001> texture are formed when heat dissipates towards the melt pool as the center of a 45° directional relationship. In other words, controlling the scanning strategy will be able to change the texture aspect. It is commonly known that if an FCC alloy develops <101> texture, it will have a greater strength and elongation compared to <001> texture^45^. Therefore, it is anticipated that strong <001> texture control with scanning strategy control will have a positive influence in improving the mechanical properties of the present alloy.

**Figure 4:**
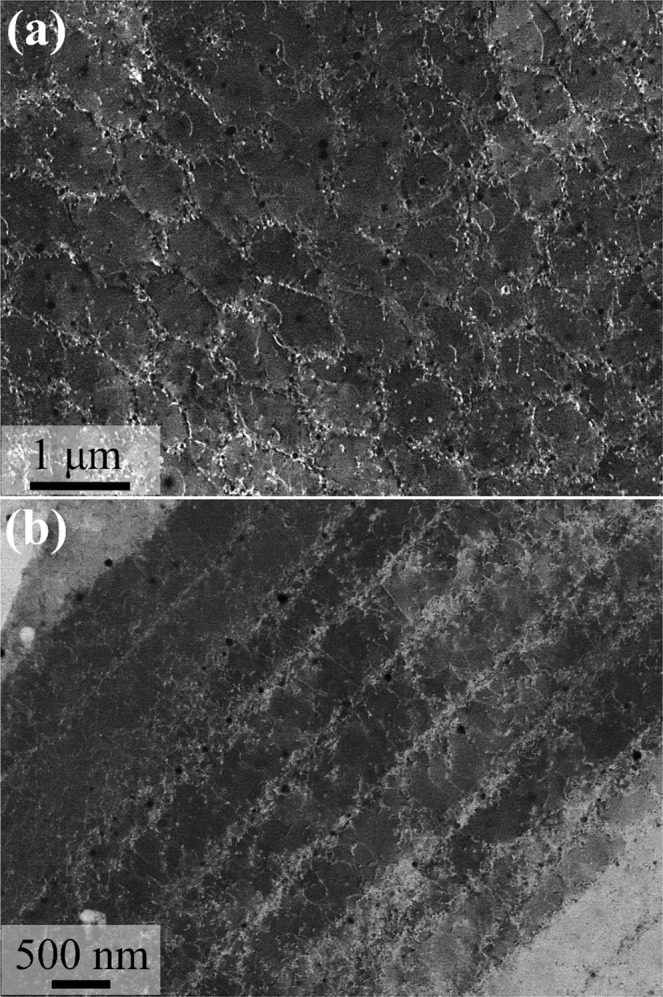
Three-dimensional EBSD IPF//BD maps of selective laser melted equiatomic CoCrFeMnNi high-entropy alloy.

AM-processed steels^49,50^, Ni-based alloys^51,52^, Al-based alloys^53,54^ and composites^55,56^ are known to form a cellular or columnar structure within the grain. The substructure formed is known to be caused by a dislocation network or elemental segregation^49,54,57^. In order to analyze the substructure within the grains of SLM-built equiatomic CoCrFeMnNi HEA, electron channeling contrast (ECC) imaging was used, and the results are shown in Fig. 4a,b. The ECC images obviously show the cellular and columnar structures in the grain, and it is suspected that these structures have a specific crystallographic orientation. In other words, a columnar structure develops in the direction the grain grows, and a cellular or columnar structure is observed depending on the cutting point (a columnar structure is formed when it is parallel and a cellular structure is formed when it is perpendicular). Also, as shown in the ECC image, cellular and columnar structures are decorated with dislocation networks, and the size measured approximately ~400 nm. This dislocation network can be understood as the compensation for distortions formed within the grain as a result of thermal contraction stress during fast cooling. In addition, scanning TEM (STEM)-EDS mapping of the cell structure confirmed that elemental segregation did not occur, and this leads to the conclusion that substructures in SLM-built HEA are only formed by dislocation networks (Fig. S2).

**Figure 5:**
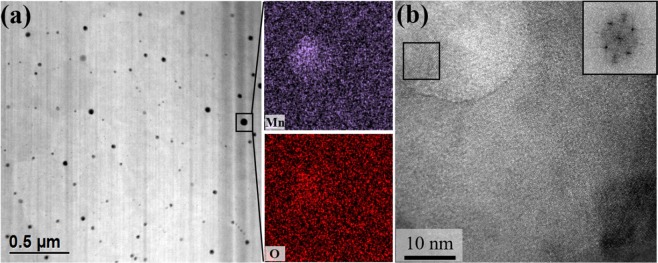
Electron channeling contrast images showing the (**a**) cellular structure and (**b**) columnar structure in the as-built sample.

Focusing on the substructure boundary in the ECC image reveals phases in the tens of nanometers with high number density. STEM-EDS mapping and high-resolution TEM were performed to analyze these nano-precipitates, and the results are shown in Fig. 5. As identified in the STEM image (Fig. 5a), phases with an average size of 27.3 nm formed in the cell structure boundary. EDS mapping confirmed it as an Mn-based oxide, and no additional phases consisting of Co, Cr, Fe and Ni were formed. To analyze this Mn-based oxide, HR-TEM and corresponding fast Fourier transform (FFT) and inverse FFT (IFFT) were performed. FFT and IFFT analysis of Mn-based oxide confirmed it to be an Mn_2_O_3_ phase with (431) and (222) planes, and the d-spacing of each plane measured 0.184 and 0.272 nm, respectively (Fig. 5b)^33^. In general, equiatomic CoCrFeMnNi HEA is known to form a Cr-Mn-based oxide or σ-phase of a few μm in size^58,59^. However, SLM-built HEA did not form a Cr-Mn-based oxide or σ-phase, which can partially hinder the mechanical properties of the alloy, and only Mn_2_O_3_ tens of nm in size was identified.

### Temperature-dependent mechanical properties

**Figure 6:**
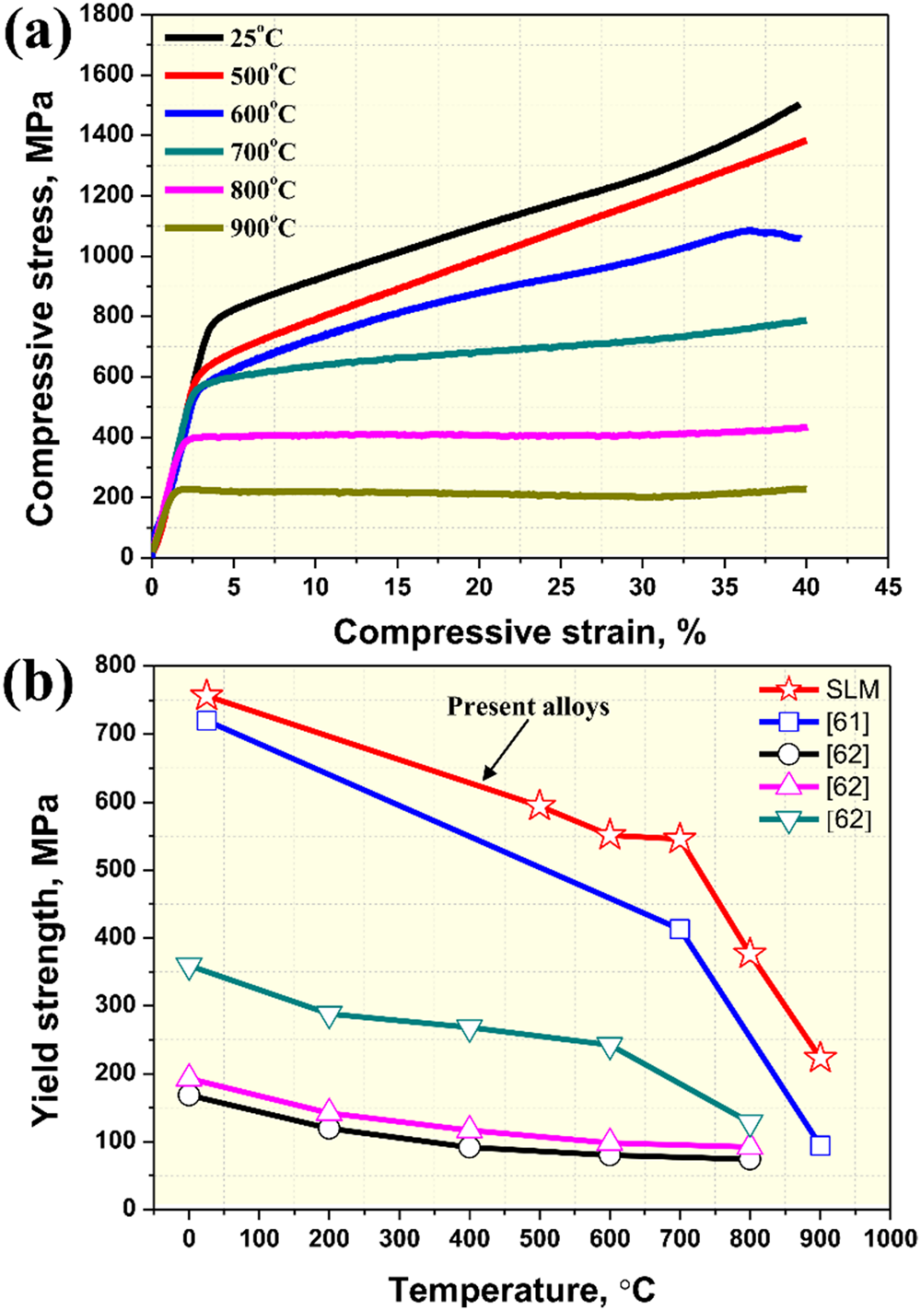
(**a**) STEM image and corresponding Mn-O elemental distribution maps and (**b**) HR-TEM images and FFT pattern of the selected square region in the HR-TEM image.

Figure 6 shows room- and high-temperature compressive stress-strain curves (a) and temperature dependence of yield strengths (b). The yield strengths (YSs) of the SLM-built equiatomic CoCrFeMnNi HEA at various temperatures are shown in Table 1. Compression tests (Fig. 6a) were performed, and the room temperature compressive yield strength measured 778.4 MPa. Compared to annealed CoCrFeMnNi HEA, which has 254.2 MPa^60^, and AM-processed CoCrFeMnNi, which has ~519 MPa^36–41^, the present alloy has relatively superior mechanical properties. The main reason why SLM-built HEA has outstanding mechanical properties is suspected to be due to the dislocation network, fine grain size and nano-sized Mn_2_O_3_ that were observed in the initial microstructure.

**Table 1:** Yield strengths (YSs) of the SLM-built equiatomic CoCrFeMnNi HEA at various temperatures.

Temperature, °C	Yield strength, MPa
25	756.5
500	594.4
600	551.3
700	545.8
800	376.6
900	223.0

SLM-built HEA showed a general tendency of decreasing strength as the compressive temperature increased. However, the yield strength for each temperature (Fig. 6b) decreased as temperature increased, but the decrease up to 700 °C was significantly smaller. In other words, SLM-built equiatomic CoCrFeMnNi HEA has superior mechanical properties up to 700 °C. Compared to CoCrFeMnNi HEAs manufactured with other manufacturing processes, the SLM-built HEA has higher mechanical properties in room temperature as well as high temperature ranges^61,62^. This means that manufacturing HEA using SLM will not only be able to produce near net shape parts, but also enable the tuning of an alloy to a strong one.

**Figure 7:**
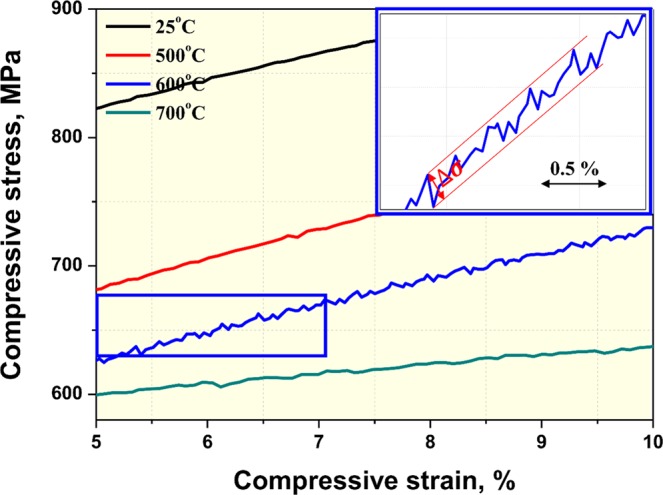
(**a**) Typical compressive stress-strain curves and (**b**) yield strengths at various temperatures.

Figure 7 shows a partially enlarged image of compressive stress strain curves of SLM-built equiatomic CoCrFeMnNi HEA. Compared to other temperatures, the 600 °C compressive stress-strain curve, remarkably, showed type-C serration. Here, type-C serration is a behavior that occurs due to load drop when deformation occurs with a low strain rate at high temperature, and the stress drop amplitude ($\triangle {\rm{\sigma }}$) was measured as ~4.5 MPa. In general, type-C serration is known to commonly occur at high temperatures as a result of the unlocking of aged dislocations^63^. The major causes of aged dislocation are i) interstitial solute atoms, ii) precipitates, and iii) substitutional solute atoms. In commercial metals and alloys, interstitial solute atoms such as C or N diffuse in this dislocation, causing dislocation moving to be suppressed, and serration occurs as a result. During this process, a solute atmosphere, which suppresses dislocation moving, is formed. And then, when the flow stress overcomes the threshold value to break this atmosphere, stress drop occurs, and the dislocations can move simultaneously. Furthermore, in cases where precipitates are formed, a serrated flow can also occur because of the interaction between the dislocation and fine precipitates^64^. It is further known that serration in substitutional solution systems can occur as a result of the interaction between solutes and dislocation motion^65^. Carroll *et al*.^66^ reported that dislocation pinning occurs due to the large number of substitutional solutes present in the whole solute matrix of FCC-based HEA. Therefore, to identify which of the three serration mechanisms has the most influence on SLM-built equiatomic CoCrFeMnNi HEA deformation at 600 °C, activation energy was analyzed (see Fig. S3). Activation energy measured 335 kJ/mol, which was significantly higher than the activation energy related to the diffusion of common interstitial solutes (76–103 kJ/mol^67^). This was also similar to or higher than the activation energies for the self-diffusion of Co, Cr, Fe, Mn and Ni, which are 270, 313, 309, 272 and 304 kJ/mol, respectively. In other words, the serrated flow of SLM-built HEA can be uninfluenced by interstitial atoms, and this is related to the cooperative lattice diffusion of constituent elements. In addition, the reason why the activation energy is higher than the self-diffusion activation energies of elements is suspected to be due to nano-sized Mn_2_O_3_, which can cause a serrated flow, also having an influence on deformation.

**Figure 8:**
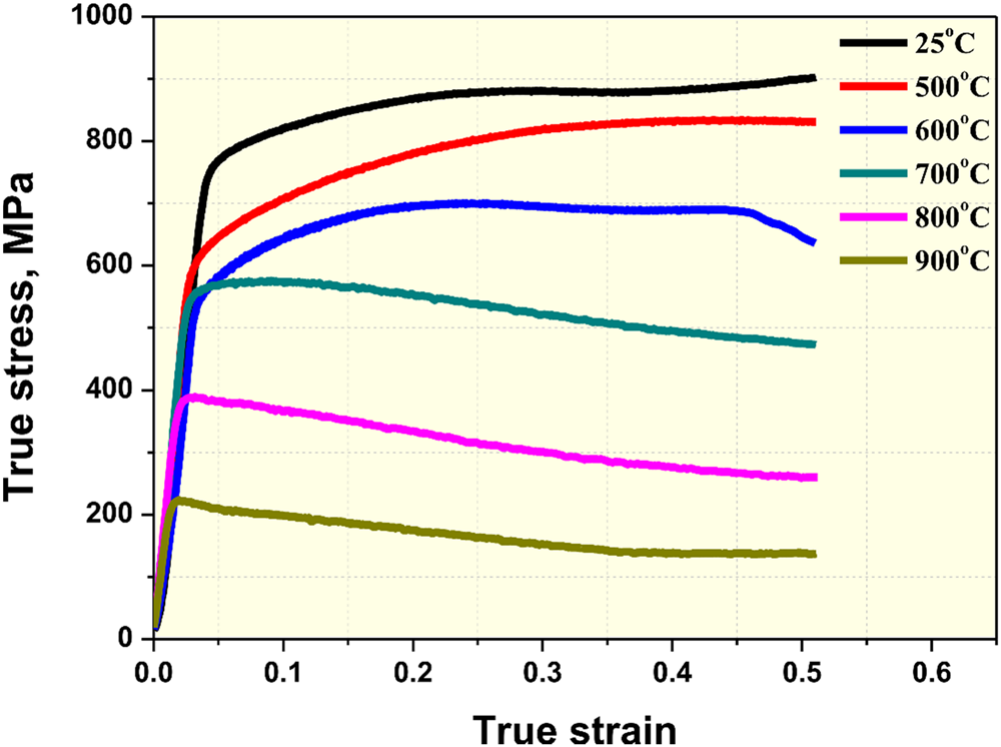
Enlarged compressive stress-strain curves showing the serrated flow.

To analyze the deformation behavior, a true-stress curve of SLM-built HEA was generated (Fig. 8). In the true stress-strain curves, work hardening continued from room temperature up to 600 °C, whereas work softening occurred at temperatures higher than 700 °C. Also, yield strength and flow stress reduction were greater at temperatures higher than 700 °C. This can be attributed to microstructure evolution (dynamic recovery or dynamic recrystallization) occurring during deformation.

### Microstructural evolution after high temperature deformation

**Figure 9:**
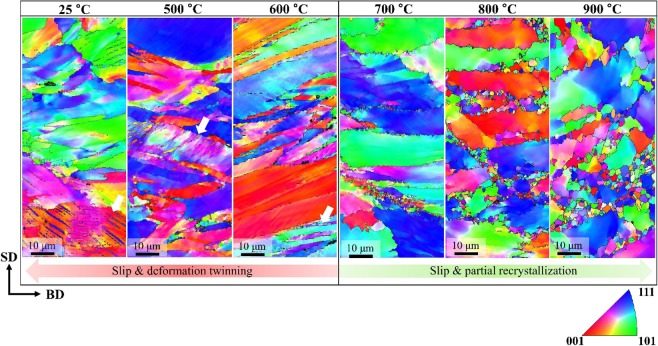
True stress-strain curves of selective laser melted equiatomic CoCrFeMnNi high-entropy alloy at various temperatures.

To understand the deformation mechanisms of SLM-built equiatomic CoCrFeMnNi HEA, the typical deformed microstructures were observed by EBSD and are shown in Fig. 9. In EBSD IPF maps at a macro strain of 40%, a large number of deformation twins (DTs, $\theta \,\approx$ 60°) were observed at room temperature. A large number of DTs (white arrows) were also observed at 500 °C and 600 °C deformations, and additional microstructure evolution (e.g., dynamic recrystallization (DRX), etc.) did not occur. On the other hand, grains of hundreds of nm up to a few μm were observed at the grain boundary at 700 °C or higher, the temperature where yield strength drop occurred. In general, such fine grains are known to be formed by DRX. So, it can be expected that the dramatic decrease in flow stress above 700 °C is due to DRX. In addition, while elongated grains were observed at 700 °C, at higher temperatures, microstructure evolution (DRX) takes place, leading to a gradual change towards equiaxed grains. Based on the above findings, it was confirmed that the major deformation mechanisms were slip and deformation twinning that occurred from room temperature up to 600 °C, and slip and partial recrystallization that occurred at temperatures higher than 700 °C.

**Figure 10:**
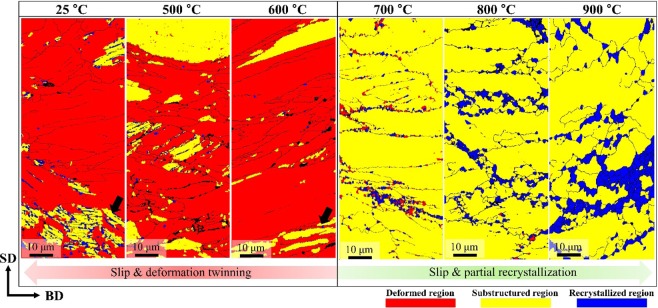
Typical EBSD IPF maps showing the deformation microstructure of SLM-built equiatomic CoCrFeMnNi high-entropy alloy.

Figure 10 shows recrystallized fraction (RF) maps of SLM-built equiatomic CoCrFeMnNi HEA at various deformation temperatures. In the case of room temperature to 600 °C samples where only work hardening occurred, deformed regions were observed in most samples, and there were some with substructured regions. In the case of 700 °C or higher samples, where yield strength drop and work softening occurred, substructured and recrystallized regions were observed in most samples. In other words, deformations at temperatures above 700 °C are expected to undergo both DRX and dynamic recovery (DRV) at the same time.

In general, DRX is differentiated among discontinuous DRX (dDRX), continuous DRX (cDRX) and geometric DRX (gDRX)^68^. dDRX is a phenomenon that commonly occurs in metallic materials with low stacking fault energy (SFE) to medium SFE, and it occurs from new strain-free grains undergoing nucleation and growth. cDRX is a phenomenon that commonly occurs in materials with high SFE, and it occurs evenly throughout a grain as low angle boundaries (LAB) gradually transforming into high angle boundaries (HAB) as deformation progresses. gDRX is a phenomenon where grains flatten in a direction perpendicular to the loading axis and new grains are formed by grain boundary migration and rotation. Considering the characteristics described above, it is suspected to occur dDRX and gDRX during the high-temperature deformation of SLM-built HEA, simultaneously.

## Discussion

### **Effects of dislocation network and Mn**_**2**_**O**_**3**_**on temperature-dependent mechanical properties**

SLM-built equiatomic CoCrFeMnNi HEA has relatively superior mechanical properties compared to known materials manufactured with conventional manufacturing processes and additive manufacturing processes^36–41,61,62^. In addition, when comparing mechanical properties with EBM-built Ti-48Al-2Cr-2Nb gamma alloy, which is attracting great attention as a high temperature material, SLM-built HEA showed higher yield strength up to ~800 °C^69^. Furthermore, when compared to oxide dispersion strengthened steels, which are developed by cryo-milling, SLM-built HEA represented excellent high-temperature strength at temperatures greater than 700 °C^70^. In our previous study, it is confirmed that the outstanding room temperature mechanical properties of SLM-built HEA can be attributed to fine grain sizes, dislocation networks and nano-sized oxides, and based on this, the authors presented their interpretation of a multiple strengthening mechanism^33^. However, it was not known until the present that SLM-built HEA has a superior mechanical property up to 700 °C. In general, the strengthening components that have an influence on mechanical properties are matrix hardening ($\triangle {{\rm{\sigma }}}_{m}$), grain boundary strengthening ($\triangle {{\rm{\sigma }}}_{k}$), dislocation forest hardening ($\triangle {{\rm{\sigma }}}_{\rho }$) and orowan strengthening ($\triangle {{\rm{\sigma }}}_{{Or}}$). However, the contributions of temperature-sensitive $\triangle {{\rm{\sigma }}}_{m}$ and $\triangle {{\rm{\sigma }}}_{k}$ decrease significantly as a result of thermally activated mechanisms in high-temperature conditions. Therefore, $\triangle {{\rm{\sigma }}}_{\rho }$ and $\triangle {{\rm{\sigma }}}_{{Or}}$ are expected to have an influence on the outstanding mechanical properties of SLM-built HEA, and this appears to be logical considering the dislocation density (1.88 × 10^14^m ^−2^) and interparticle spacing between Mn_2_O_3_ (82.07 nm) of the SLM-built HEA. In relatively low temperature conditions (~600 °C), ${{\rm{\sigma }}}_{m}$, ${{\rm{\sigma }}}_{k}$, ${{\rm{\sigma }}}_{\rho }$ and ${{\rm{\sigma }}}_{{Or}}$ contribute to achieving mechanical properties greater than those of annealed and hot-rolled equiatomic CoCrFeMnNi HEAs. In high temperature conditions (above 700 °C), the outstanding high-temperature mechanical properties are achieved as ${{\rm{\sigma }}}_{\rho }$ and ${{\rm{\sigma }}}_{{Or}}$ have a significant influence on strengthening even though ${{\rm{\sigma }}}_{m}$ and ${{\rm{\sigma }}}_{k}$ cannot contribute due to the thermally activated mechanism.

### Deformation behavior of SLM-built equiatomic CoCrFeMnNi high-entropy alloy

**Figure 11:**
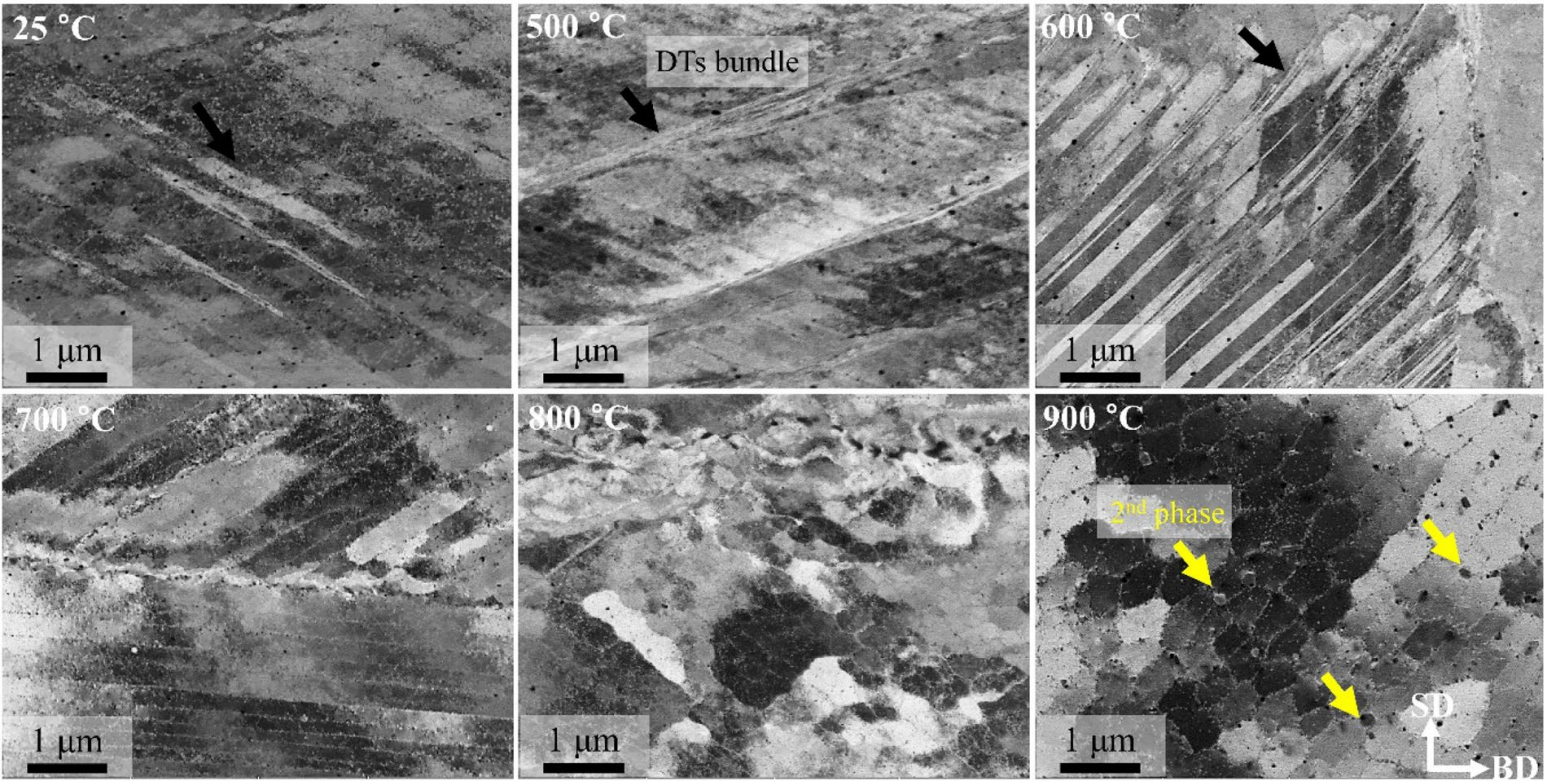
Typical EBSD RF maps showing the deformation microstructure of SLM-built equiatomic CoCrFeMnNi high-entropy alloy. The fraction of deformed, substructured, and recrystallized grains was calculated using a recrystallization map component in Tango after keeping a minimum misorientation angle of 2 deg. to separate sub-grains and 15 deg. to separate grains.

Figure 11 shows ECC images at each temperature to analyze the room to high temperature deformation behavior of SLM-built HEA. Like the EBSD analysis, a large number of DT bundles was identified from room temperature to 600 °C, and this confirms that the deformation mechanisms in low temperature conditions are slip and DT evolution. In the range of temperatures above 700 °C, the deformed samples had a clear cellular and columnar structure composed of dislocation networks as well as nano-sized oxides, and this confirmed that the slip and partial RX observed in EBSD are the main deformation mechanisms. In addition, the reason why work hardening occurs at temperatures below 600 °C is suspected to be because dislocation is suppressed as a result of dislocation density increase and DT evolution. In contrast, above 700 °C, the substructure is maintained due to dislocation climb, and yield strength drop and work softening are suspected to occur due to partial RX. In the case of the sample deformed at 900 °C, σ-phase was formed at the cell structure boundary.

Up to the present, RX occurring during the high-temperature deformation of equiatomic CoCrFeMnNi HEA was mainly explained as dDRX^71^. However, Fig. 9 shows flattened grains formed perpendicular to the loading axis, and a large number of grain boundary serrations (migration of grain boundary) are found. This is a phenomenological characteristic of gDRX, and based on this finding, gDRX is also occur simultaneously in high-temperature deformation of SLM-built HEA with dDRX. The same evidence can be found in the true stress-strain curve of Fig. 9. As seen in the curve, gDRX, where flow stress continuously decreases by small amounts, is somewhat different with dDRX, where flow stress decreases sharply, and cDRX, where saturation occurs (See Fig. S4).

**Figure 12:**
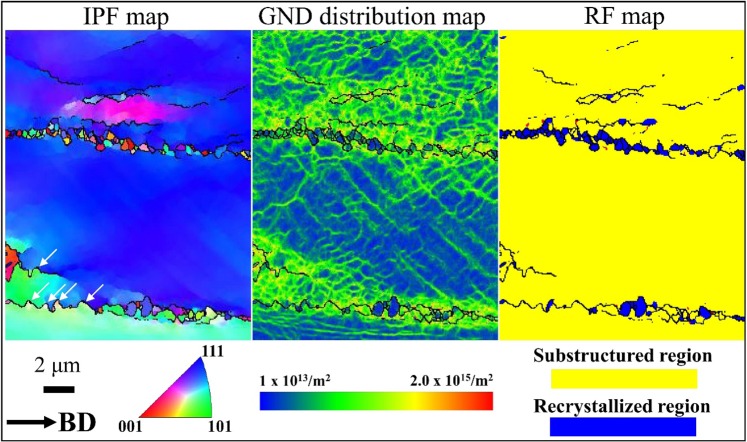
ECC images of SLM-built HEAs after compressive deformation at various temperatures.

In order to analyze the DRX phenomenon, HR-EBSD analysis was performed on the sample deformed at 700 °C (Fig. 12). Here, (a) is an IPF map, (b) is a geometrically necessary dislocation (GND) distribution map and (c) is a RF map. In the IPF map, ultra-fine grains with an average size of 378 nm were found at grain boundaries. On the other hand, the migration of HABs was observed in grain boundaries with no ultra-fine grains (white arrow). In the GND distribution map, the dislocations in DRXed grains confirmed that they are not new, strain-free grains (i.e., the main RX mechanism in dDRX), and it is possible to expected that ultra-fine grains are formed due to the rotation of HABs. In other words, the DRX of SLM-built HEA is caused by combination of dDRX and gDRX, which follow the process of i) accumulation of dislocations at HABs, ii) migration or bulging of HABs, and iii) rotation of HABs.

On the other hand, the reason why a dramatic decrease in flow stress did not occur in SLM-built equiatomic CoCrFeMnNi HEA deformations up to 900 °C is suspected to be due to full recrystallization not occurring easily. This is suspected to be a result of the Zener drag effect^72^ caused by the oxides formed at the cellular structure boundary and grain boundary, which delay RX as a result. In general, the overall pressure ($P$) for recrystallization is expressed as the following Formula 1:

1 $$P={P}_{D}-{P}_{Z}=3{\gamma }_{s}/D-3{F}_{v}{\gamma }_{b}/d$$

where *P*_*D*_ is driving pressure for recrystallization, *P*_*Z*_ represents Zener pinning pressure, γ_*s*_ is sub-grain boundary energy, *D* is sub-grain diameter, *F*_*v*_ is volume fraction of particles, γ_*b*_ is high angle grain boundary energy, and $d$ is particle diameter. As SLM-built equiatomic CoCrFeMnNi HEA has a high fraction *F*_*v*_ and $d$ of 27.3 nm, *P*_*Z*_ increases significantly. Therefore, the overall pressure for RX decreases dramatically. In addition, the migrated grain boundary formed during gDRX is also a factor that delays RX. If a migrated grain boundary with a radius of *R* is present, further retarding pressure (*P*_*C*_) occurs, and this is also known to delay DRX^73^. Therefore, while *P*_*D*_ is identical, the overall pressure for recrystallization decreases as a result of the Zener pinning pressure (*P*_*Z*_) caused by Mn_2_O_3_ and further retarding pressure (*P*_*C*_) caused by the migrated grain boundary. As RX is delayed due to this, SLM-built equiatomic CoCrFeMnNi HEA is expected to delay strain softening up to 900 °C.

## Conclusions

Correlations among the microstructure, temperature-dependent mechanical properties, and deformation behavior of selective laser-melted equiatomic CoCrFeMnNi high-entropy alloy were investigated. The following conclusions were drawn from the results of this study:

1. SLM-built equiatomic CoCrFeMnNi HEA is confirmed to have a random solid solution FCC single phase. In addition, epitaxial growth grains were identified, and the average grain size of BD, TD, and SD planes measured 5.98 μm, 12.93 μm and 15.66 μm, respectively. Cellular and columnar structures composed of dislocation networks were formed within the grain, and Mn_2_O_3_ of 27.3 nm was found at the interface.
2. SLM-built equiatomic CoCrFeMnNi HEA was confirmed to have outstanding mechanical properties in room to high temperature ranges. Fine grain size, dislocation network and nano-sized oxides are suggested to be contributing factors, and in high temperature conditions, dislocation forest hardening and Orowan strengthening were confirmed to be the major strengthening mechanisms. In addition, type-C serration occurred at 600 °C deformation, and this is expected to be due to the interaction between constituent elements and dislocation.

Compressive deformation microstructure observation confirmed that slip and DT were the dominant behaviors from room temperature to 600 °C, and slip and DRX were confirmed to be the major deformation mechanisms above 700 °C. Furthermore, an extreme flow stress decrease does not occur in SLM-built HEA up to a condition of ~900 °C, and this is suspected be the result of a decrease in driving force of recrystallization caused by nano-sized oxides and migrated grains.

## Methods

### Powder material and SLM manufacturing conditions

**Figure 13:**
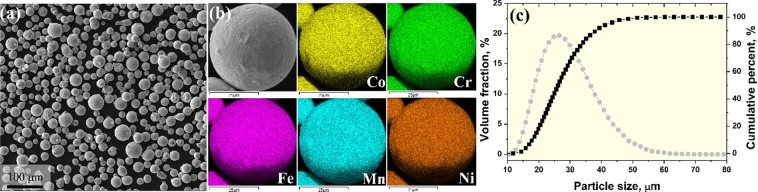
EBSD IPF map (**a**), GNDs distribution maps (**b**), and RF map of deformed sample at 700 °C.

Figure 13 shows a scanning electron microscopy (SEM) image (Fig. 13a), EDS mapping image (Fig. 13b), and particle size distribution (Fig. 13c) of the pre-alloyed equiatomic CoCrFeMnNi HEA powder. The pre-alloyed equiatomic CoCrFeMnNi powder had a spherical shape, and was confirmed to have an even distribution of Co, Cr, Fe, Mn, and Ni elements. The average diameter of the initial powders was measured as 27.2 um (*d*_10_ = 17.9 µm, *d*_50_ = 26.1 µm, *d*_90_ = 38.2 µm). Also, EBSD analysis of the initial powder measured an average powder particle grain size of 3.2 µm (Fig. S5).

The above powders were then used as feedstocks for SLM processing (model: Concept Laser Mlab cusing) under high-purity Ar gas to minimize oxidation. A laser scanning speed of 600 mm/s was applied to fabricate bar-type specimens with dimensions of 85 mm × 10  m × 10  m. A hatch space of 80 µm with a laser power of 90 W was applied. The layer thickness was set to 25 µm. In addition, a scanning pattern of 180° angle rotation for the two consecutive layers was applied. Volumetric energy density (VED) is considered a key factor for the microstructure and properties of SLM-processed materials, and is defined as follows^38^:

2 $$VED=\frac{P}{vht}$$

where *t* denotes layer thickness, *v* is the scan speed, *P* is the laser power, and *h* is the hatch space. The VED was measured to 75 J/mm^3^ in the present study.

### Microstructural characterization

To identify the phase of SLM-built HEA, an X-ray diffractometer (XRD, Ultima IV, Cu K_α_ radiation, scan step size; 0.02 deg., scan rate; 2 deg. min^−1^) was used. The average dislocation density of as-built equiatomic CoCrFeMnNi HEA was calculated using the convolutional multiple whole profile (CMWP) method^74^. To observe the initial and compressive deformed microstructures of SLM-built equiatomic CoCrFeMnNi HEA, samples were ground using silicon carbide papers with #400~#1200 grit, and 1 μm diamond suspension, and then mirror polished more using a colloidal silica (CS) suspension. Back-scattered electron imaging (BSEI) was conducted with field emission scanning electron microscopy (FE-SEM, MYRA 3 XMH, Tescan, Czech Republic). In addition, crystallographic orientation and grain size were analyzed by electron backscatter diffraction (EBSD, Nordlys-CMOS detector, binning: 2 × 2, step size: (1 μm (Figs. 2 and 3), 120 nm (Figs. 9 and 10) and 30 nm (Fig. 12)), Oxford, United Kingdom). Dislocation and nano-sized particle distributions were analyzed by electron channeling contrast (ECC) imaging at an acceleration voltage of 30 kV with a BSE detector. The working distance of ECCI was set to 7 mm with a sample tilt of 2.6°. To analyze nano-sized precipitates in SLM-built equiatomic CoCrFeMnNi, a Cs-corrected scanning transmission electron microscope (STEM, JEM-ARM200F, Japan) and field emission TEM (FE-TEM, JEM-2100F, Japan) were used. The density of the SLM-built HEA was calculated by Archimedes’ principle.

### Room and high temperature compression testing

To evaluate the temperature-dependent mechanical properties of SLM-built equiatomic CoCrFeMnNi, a cylindrical specimen with a size of Φ 4 mm × height 6 mm was prepared by using electrical discharge machining according to scanning direction (SD). Compression tests were performed using MTS-810 equipment at 25, 500, 600, 700, 800, and 900 °C. An initial strain rate of 1 × 10^−3^  ^−1^ and an engineering strain limit of 0.4 were applied. Before the temperature-dependent compressive tests, the SLM-built equiatomic CoCrFeMnNi HEAs were preheated in an electric resistance furnace at a heating rate of 10 °C/min. The compression tests were conducted three times for each condition. Furthermore, MoS_2_ spray used as a lubricant to reduce physical friction.

## Supplementary information

Supplementary information.
