# Author Correction: Superior Temperature-Dependent Mechanical Properties and Deformation Behavior of Equiatomic CoCrFeMnNi High-Entropy Alloy Additively Manufactured by Selective Laser Melting

**DOI:** 10.1038/s41598-020-69894-z

**Published:** 2020-10-19

**Authors:** Young-Kyun Kim, Sangsun Yang, Kee-Ahn Lee

**Affiliations:** 1 Department of Materials Science and Engineering, Inha University, Incheon, 22212 Republic of Korea; 2 Korea Institute of Materials Science (KIMS), Changwon, 51508 Republic of Korea

Correction to: *Scientific Reports* https://doi.org/10.1038/s41598-020-65073-2, published online 15 May 2020

This Article contained errors in the order of the Figure legends.

The Figure 1 legend was incorrectly given as the Figure 2 legend; The Figure 2 legend was incorrectly given as the Figure 3 legend; The Figure 3 legend was incorrectly given as the Figure 4 legend; The Figure 4 legend was incorrectly given as the Figure 5 legend; The Figure 5 legend was incorrectly given as the Figure 6 legend; The Figure 6 legend was incorrectly given as the Figure 7 legend; The Figure 7 legend was incorrectly given as the Figure 8 legend; The Figure 8 legend was incorrectly given as the Figure 9 legend; The Figure 9 legend was incorrectly given as the Figure 10 legend; The Figure 10 legend was incorrectly given as the Figure 11 legend; The Figure 11 legend was incorrectly given as the Figure 12 legend; The Figure 12 legend was incorrectly given as the Figure 13 legend; The Figure 13 legend was incorrectly given as the Figure 1 legend.

The correct order of the Figure Legends is listed below:

**Figure 1**. Elemental distribution analysis results of as-built equiatomic CoCrFeMnNi high-entropy alloy using back scattered electron (BSE) – energy dispersive X-ray spectroscopy (EDS) mapping.

**Figure 2**. (**a**) X-ray diffraction (XRD) patterns and (**b**) EBSD phase map of as-built sample (HAGB: high angle grain boundary).

**Figure 3**. Three-dimensional EBSD IPF/BD maps of selective laser-melted equiatomic CoCrFeMnNi high-entropy alloy.

**Figure 4**. Electron channeling contrast images showing (**a**) the cellular structure and (**b**) the columnar structure in the as-built sample.

**Figure 5**. (**a**) Scanning transmission electron microscopy (STEM) image and corresponding Mn-O elemental distribution maps and (**b**) high resolution TEM (HR-TEM) images and FFT pattern of the selected square region in the HR-TEM image.

**Figure 6**. (**a**) Typical compressive stress-strain curves and (**b**) yield strengths at various temperatures.

**Figure 7**. Enlarged compressive stress-strain curves showing the serrated flow.

**Figure 8**. True stress-strain curves of selective laser-melted equiatomic CoCrFeMnNi high-entropy alloy at various temperatures.

**Figure 9**. Typical EBSD IPF maps showing the deformation microstructure of SLM-built equiatomic CoCrFeMnNi high-entropy alloy.

**Figure 10**. Typical EBSD RF maps showing the deformation microstructure of SLM-built equiatomic CoCrFeMnNi high-entropy alloy. The fraction of deformed, substructured, and recrystallized grains was calculated using a recrystallization map component in Tango after keeping a minimum misorientation angle of 2 deg. to separate sub-grains and 15 deg. to separate grains.

**Figure 11**. ECC images of SLM-built HEAs after compressive deformation at various temperatures.

**Figure 12**. EBSD IPF map (**a**), GNDs distribution maps (**b**), and RF map of deformed sample at 700 °C.

**Figure 13**. Raw equiatomic CoCrFeMnNi HEA pre-alloyed powders: (**a**) SEM morphology, (**b**) SEM-EDS mapping results, and (**c**) particle size distributions.

These errors have now been corrected in the HTML and PDF version of the Article.

