# Supplementary material for: Superior Temperature-Dependent Mechanical Properties and Deformation Behavior of Equiatomic CoCrFeMnNi High-Entropy Alloy Additively Manufactured by Selective Laser Melting
Source: Sci Rep. 2020 May 15;10:8045. doi: 10.1038/s41598-020-65073-2 (PMC7229066; doi:10.1038/s41598-020-65073-2)
Supplement: Supplementary file 1 — Supplementary information. [file 41598_2020_65073_MOESM1_ESM.docx]

**Supplementary material**

**Superior Temperature-Dependent Mechanical Properties and**

**Deformation Behavior of Equiatomic CoCrFeMnNi High-Entropy Alloy**

**Manufactured by Selective Laser Melting**

Young-Kyun Kim^1^, Sangsun Yang^2^, Kee-Ahn Lee^1,*^

^1^Department of Materials Science and Engineering, Inha University, Incheon 22212, Republic of Korea

^2^Korea Institute of Materials Science (KIMS), Changwon 51508, Republic of Korea

**^*^**Corresponding author: Kee-Ahn Lee, keeahn@inha.ac.kr

**
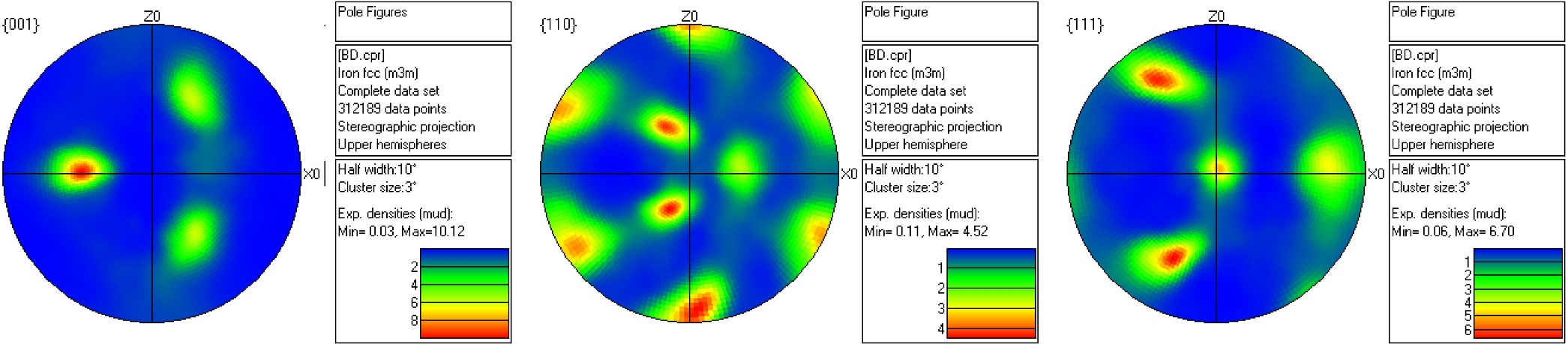
**

**Fig. S1.** EBSD pole figure analysis results for BD of as-built HEA.

**
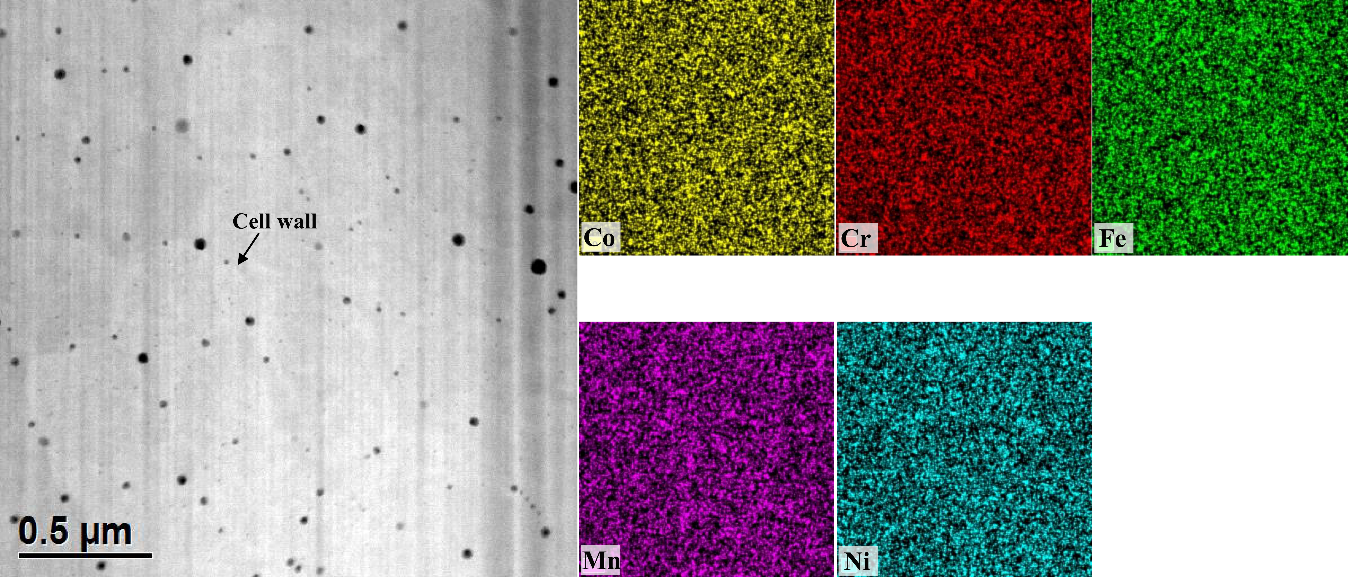
**

**Fig. S2.** STEM-EDS mapping results of SLM-built equiatomic CoCrFeMnNi high-entropy alloy.


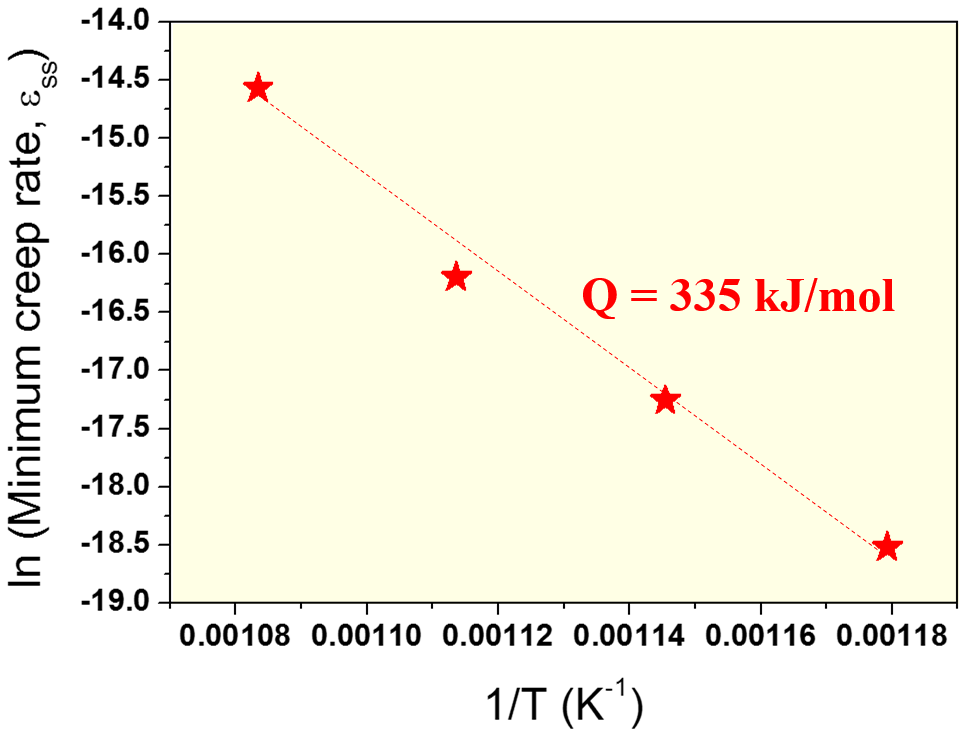


**Fig. S3.** Arrhenius plot for determining activation energy.


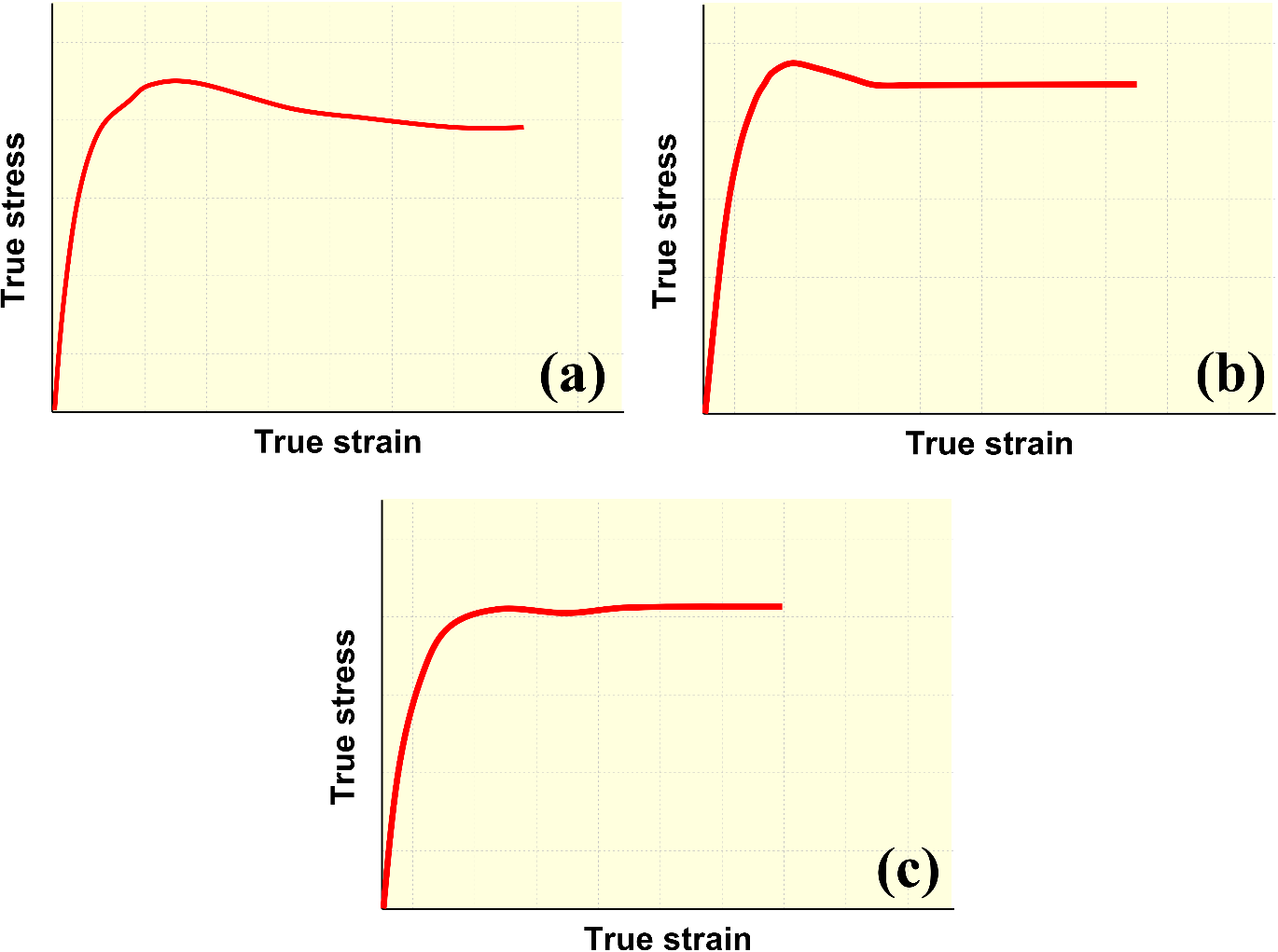


**Fig. S4.** Redrawn schematic illustration of the typically observed experimental stress-strain response [1]; (a) gDRX, (b) dDRX, and (c) cDRX.

**
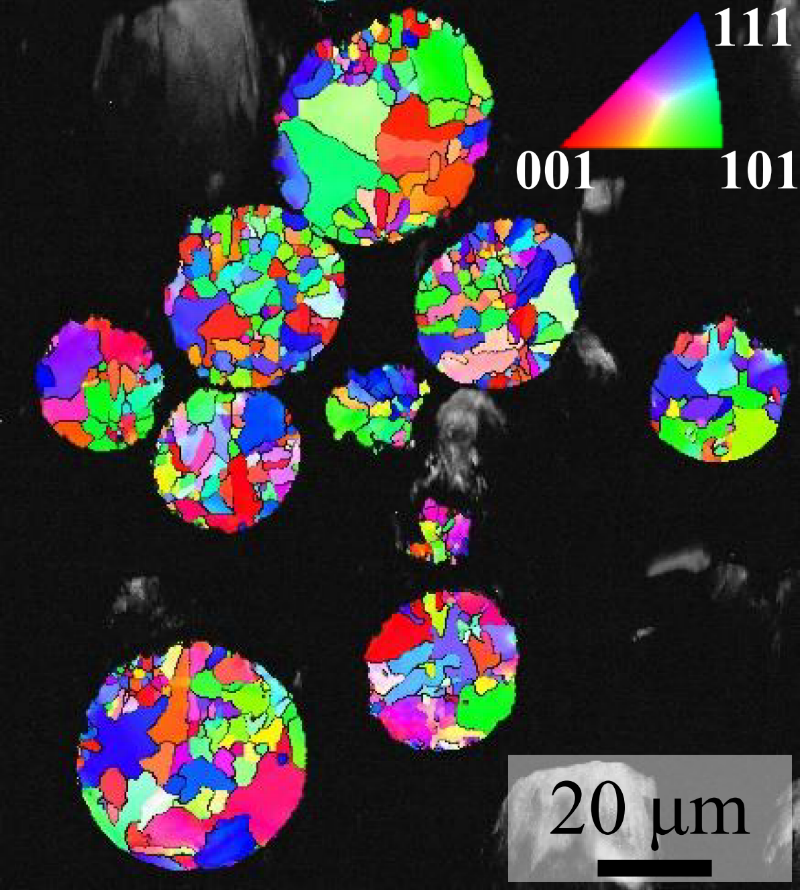
**

**Fig. S5.** EBSD inverse pole figure maps of pre-alloyed powders.

**Reference**

[1] K. Huang, R.E. Loge, A review of dynamic recrystallization phenomena in metallic materials, Mater. Des. 111 (2016) 548-574.
